# Comparative In Vitro Evaluation of the Remineralization Potential of Commercial Pediatric Dentifrices Containing Herbal Ingredients (Ginger, Propolis, Aloe Vera, and Rosemary), Fluoride, and CPP-ACP on Primary Enamel: An SEM-EDX Study

**DOI:** 10.3390/jfb17100494

**Published:** 2026-09-30

**Authors:** Hayriye Zehra Türk, Ebru Akleyin

**Affiliations:** Department of Pediatric Dentistry, Faculty of Dentistry, Dicle University, 21300 Diyarbakır, Türkiye; turkzehra03@gmail.com

**Keywords:** primary tooth enamel, remineralization, commercial dentifrices, fluoride, CPP-ACP, herbal dentifrices, SEM-EDX

## Abstract

**Objective:** The aim of this study was to comparatively evaluate the effects of fluoride- and casein phosphopeptide–amorphous calcium phosphate (CPP-ACP)-containing products and commercial pediatric dentifrices containing herbal ingredients (propolis, ginger, aloe vera, and rosemary) on artificially induced initial caries lesions in primary tooth enamel using scanning electron microscopy (SEM) and energy-dispersive X-ray spectroscopy (EDX). **Materials and Methods:** Eighty caries-free primary molars were randomly allocated into eight groups (*n* = 10): two control groups (sound and demineralized enamel), a fluoride group, a CPP-ACP group, and four commercial herbal dentifrice groups containing propolis, ginger, aloe vera, or rosemary. Artificial initial enamel lesions were created in all specimens except those in the positive control group. During a 7-day pH-cycling protocol, the treatment groups received standardized dentifrice slurry applications twice daily for 4 min. At the end of the experimental period, enamel surfaces were evaluated using SEM-EDX. Statistical analyses were performed using one-way analysis of variance (ANOVA) and the Kruskal–Wallis test, as appropriate (*p* < 0.05). **Results:** SEM analysis revealed a comparatively compact and homogeneous surface appearance in the fluoride group, while the ginger group showed a relatively homogeneous surface morphology among the commercial herbal dentifrice groups. EDX analysis showed no statistically significant differences among the groups in Ca wt% (*p* = 0.085), Ca at% (*p* = 0.077), atomic Ca/P ratio (*p* = 0.138), or weight Ca/P ratio (*p* = 0.126). Significant between-group differences were observed for both P wt% (*p* = 0.008) and P at% (*p* = 0.005), as well as O wt% (*p* = 0.022) and O at% (*p* = 0.020). **Conclusions:** The tested products produced qualitative differences in enamel surface micromorphology and significant differences in some EDX-derived relative elemental percentages; however, Ca percentages and Ca/P ratios did not differ significantly among the groups. Therefore, the observed numerical and morphological variations should not be interpreted as evidence of superior mineral gain or remineralization efficacy. Further in situ and in vivo studies are required to determine the clinical relevance of these findings.

## 1. Introduction

Dental caries is a dynamic and multifactorial disease that develops as a result of the metabolism of dietary fermentable carbohydrates by biofilm microorganisms, leading to progressive mineral loss from dental hard tissues [1]. During early childhood, the thinner enamel structure and lower mineral density of primary teeth may accelerate the development and progression of early childhood caries (ECC) [2]. Under physiological conditions, demineralization and remineralization exist in a dynamic equilibrium; however, when plaque pH falls below the critical threshold, ions begin to dissolve from enamel hydroxyapatite crystals, resulting in the development of initial non-cavitated enamel lesions (white spot lesions) [3]. As surface integrity is preserved in these initial lesions, they may be reversed through non-invasive remineralization approaches [4].

Dentifrices, which facilitate mechanical plaque removal, are complex formulations composed of active and inactive auxiliary ingredients [5]. In pediatric dentifrices, safety is as important as clinical efficacy; because of the tendency toward accidental or reflexive swallowing, low abrasivity, the use of non-cariogenic sweeteners, and the age-appropriateness of the incorporated agents are important considerations [6]. Fluoride has long been regarded as the gold standard because of its ability to enhance enamel resistance to acid challenge [7], while biomimetic materials such as casein phosphopeptide–amorphous calcium phosphate (CPP-ACP) provide a highly bioavailable source of mineral ions [8]. Although fluoride remains the gold standard for caries prevention, excessive fluoride exposure during tooth development may increase the risk of dental fluorosis, highlighting the importance of age-appropriate fluoride use in children [6].

In this context, herbal dentifrices containing natural extracts such as propolis, ginger, aloe vera, and rosemary have attracted increasing attention in preventive pediatric dentistry due to their broad-spectrum antimicrobial, antioxidant, and anti-inflammatory properties [9]. These agents have been reported to exhibit antimicrobial effects, including inhibition of biofilm formation and *Streptococcus mutans* colonization, while some formulations have also been associated with increased microhardness and reduced demineralization [10]. Although their natural origin and low adverse-effect profiles offer important advantages, variability in raw materials and difficulties in pharmaceutical standardization remain major limitations [11]. Recent evidence has also highlighted the increasing use of biomimetic and nano-hydroxyapatite-based approaches for enamel remineralization [12,13]. Importantly, the assessment of remineralization may involve complementary structural, chemical, and mechanical methods, as individual analytical techniques provide information on different aspects of enamel mineral recovery [14].

Previous studies have evaluated the effects of herbal-containing dentifrices, fluoride, and CPP-ACP on enamel demineralization and remineralization through various comparisons; however, studies simultaneously comparing commercial multi-ingredient formulations containing these components within the same experimental model in primary tooth enamel remain limited [15,16,17]. For the assessment of remineralization-related changes, scanning electron microscopy (SEM) provides qualitative information on enamel surface morphology, while energy-dispersive X-ray spectroscopy (EDX) provides information on relative elemental composition (Ca, P, O, and the Ca/P ratio) within the analyzed surface regions [18].

Ginger, propolis, aloe vera, and rosemary were selected for the present study because their strong antimicrobial and antioxidant properties, as well as their potential protective effects against enamel demineralization, have been reported in the literature. In addition, these herbal ingredients are widely available in commercially marketed products and are among the popular natural ingredients readily accessible to parents. Therefore, this study aimed to comparatively evaluate the effects of commercial dentifrices containing ginger, propolis, aloe vera, or rosemary, together with fluoride- and CPP-ACP-containing products, on artificially demineralized primary tooth enamel using SEM and EDX analyses.

**Null Hypothesis** **(H0).** *The null hypothesis was that there would be no significant differences among the tested dentifrice groups in the EDX-derived elemental composition and Ca/P ratios of primary enamel.*

## 2. Materials and Methods

The study protocol was approved by the Ethics Committee of Dicle University Faculty of Dentistry (2025/102, approval date: 31 December 2025). Eighty sound human primary molars extracted due to physiological root resorption and presenting no enamel defects or caries were included in the study. The specimens were stored in distilled water at 4 °C for approximately six months until the experimental procedures were initiated. After removal of soft-tissue remnants and polishing with a fluoride-free polishing paste, the crowns were separated from the roots using a diamond bur. A 2 × 2 mm experimental window was delineated with adhesive tape at the center of the buccal surface of each tooth, and all remaining exposed surfaces were isolated with two coats of acid-resistant nail varnish. The specimens were randomly assigned to eight experimental groups (*n* = 10 per group) without the use of a computer-generated randomization sequence (Figure 1):

- Group 1 (Positive Control): Sound enamel (no treatment or experimental procedure).
- Group 2 (Negative Control): Demineralized enamel (pH cycling without treatment).
- Group 3 (Fluoride): Dentifrice containing 1450 ppm fluoride (NaF) (Sensodyne Promine Kids 6–12 Years).
- Group 4 (CPP-ACP): Bioactive CPP-ACP cream (GC Tooth Mousse; GC Corporation, Tokyo, Japan).
- Group 5 (Propolis): Propolis-containing dentifrice (DK Dent, Dermokil, Sivas, Türkiye).
- Group 6 (Ginger): Ginger-containing dentifrice (Eyüp Sabri Tuncer, Lalahan, Ankara, Türkiye).
- Group 7 (Aloe Vera): Aloe vera-containing pediatric dentifrice (DK Dent, Dermokil, Sivas, Türkiye).
- Group 8 (Rosemary): Rosemary-containing dentifrice (Ormanya, Seka Park Ormanya, Kocaeli, Türkiye).

The compositions of the commercial products used in the study, as reported by the manufacturers, are presented in Table 1. Because the tested materials were commercial multi-ingredient formulations, the observed effects cannot be attributed solely to the named active ingredients (fluoride, CPP-ACP, propolis, ginger, aloe vera, or rosemary), as other components of each formulation may also have influenced the findings.

### 2.1. Artificial Initial Caries Lesion Formation and pH-Cycling Protocol

Prior to the experimental procedures, all teeth were immersed in artificial saliva for 30 min and then air-dried for approximately 5 s. A demineralization solution containing 1.5 mM CaCl_2_, 0.9 mM KH_2_PO_4_, and 50 mM acetic acid (pH 4.8) and a remineralization solution containing 1.5 mM CaCl_2_, 0.9 mM KH_2_PO_4_, 130 mM KCl, and 20 mM HEPES (pH 7.0) were prepared. Group 1 received neither solution exposure nor treatment, whereas Group 2 underwent pH cycling without the application of any remineralizing material.

To induce initial enamel lesions, specimens in all groups except Group 1 were immersed in the demineralization solution for 72 h and subsequently subjected to pH cycling at 37 °C for 7 consecutive days. The ten specimens within each group were maintained together in the same container. During each daily pH cycle, the specimens were immersed in the demineralization solution for 6 h, rinsed with distilled water for 5 s to prevent carryover between solutions, and then immersed in the remineralization solution for 18 h. The same demineralization and remineralization solutions were used throughout the 7-day pH-cycling period. The solutions were stored refrigerated and allowed to reach room temperature for several hours before use. For the experimental groups, homogeneous dentifrice slurries were prepared by mixing one part dentifrice with three parts distilled water (1:3, dentifrice:water). The fluoride concentration reported for the commercial fluoride dentifrice (1450 ppm F^−^) refers to the undiluted product; the dentifrice was applied experimentally as the 1:3 dentifrice–water slurry described above. The prepared slurries were applied to the specimens twice daily, before and after the demineralization period, for 4 min per application. For experimental standardization, the same application duration and frequency were used for all tested products, including CPP-ACP; therefore, the CPP-ACP protocol represented a standardized experimental exposure rather than the manufacturer-recommended clinical application regimen. Following each application, the specimens were rinsed with distilled water for 5 s and transferred to the corresponding stage of the pH-cycling protocol.

After completion of the pH-cycling period, all specimens were immersed for 30 min at 37 °C in an artificial saliva solution (pH 7.0) containing 0.08% NaCl, 0.12% KCl, 0.01% MgCl_2_·6H_2_O, 0.03% KH_2_PO_4_, 0.01% CaCl_2_·2H_2_O, and 99.75% distilled water. This final standardized exposure was performed to simulate the oral environment prior to analysis. The specimens were subsequently dried in an incubator at 37 °C for 24 h before SEM-EDX analysis (Table 2).

### 2.2. SEM and EDX Analysis

SEM and EDX analyses of the specimens were performed at the Dicle University Science and Technology Application and Research Center (DÜBTAM) using a field-emission scanning electron microscope equipped with energy-dispersive X-ray spectroscopy (FEG-SEM/EDX; Quanta FEG 250, FEI Company, Hillsboro, OR, USA). Prior to analysis, the specimens were coated with goldpalladium (Au-Pd) under standard vacuum conditions to ensure electrical conductivity. Au-Pd coating peaks were excluded from the quantitative EDX elemental analysis. Accordingly, the EDX-derived elemental percentages were interpreted as relative surface compositional measurements rather than absolute measures of enamel mineral content. For data processing, the Ca, P, and O weight (wt%) and atomic (at%) percentages were proportionally normalized within each specimen so that the sum of Ca, P, and O equaled 100%. The Ca/P ratios were subsequently calculated from the normalized Ca and P values.

For SEM analysis, enamel surface topography was evaluated at ×1000 magnification using an accelerating voltage of 20.0 kV, a working distance of approximately 11.7 mm, an Everhart–Thornley detector (ETD), and a horizontal field width of 207 µm. For EDX analysis, five microareas were selected within the 2 × 2 mm exposed enamel window of each specimen, and the mean of these measurements was used for the statistical analysis. The weight (wt%) and atomic (at%) percentages of calcium (Ca), phosphorus (P), and oxygen (O) were obtained from the EDX quantitative outputs. Following the normalization procedure described above, the mean values derived from the five microareas of each specimen were used for statistical analysis. The specimen-level EDX dataset used for the reported analyses is provided in Supplementary Data S1. Representative EDX spectra for each experimental group are provided in Figure S1.

### 2.3. Statistical Analysis

The sample size was determined using an a priori power analysis performed with G*Power 3.1. For the comparison of eight experimental groups, a one-way analysis of variance (ANOVA) was considered. In the absence of preliminary data suitable for estimating an empirical effect size, a conventional large effect size of f = 0.50 was selected based on Cohen’s effect size classification [19]. With eight groups, a significance level of α = 0.05, and a statistical power of 80% (1 − β = 0.80), the minimum required total sample size was calculated as 65. To ensure equal sample sizes across groups and account for potential specimen loss, 10 specimens were included in each group, resulting in a total sample size of 80.

Statistical analyses were performed using IBM SPSS Statistics version 31.0 (IBM Corp., Armonk, NY, USA). The normality of data distribution was assessed using the Shapiro–Wilk test, and homogeneity of variances was evaluated using Levene’s test. Variables meeting the assumptions of normality were compared among groups using one-way ANOVA, with post hoc comparisons performed using the Games–Howell test, taking variance homogeneity into account. For data that did not meet the assumptions of parametric testing, the Kruskal–Wallis test was used, followed by the Dunn–Bonferroni test for variables showing statistically significant differences. Effect sizes were evaluated using eta squared (η^2^) for ANOVA and epsilon squared (ε^2^) for the Kruskal–Wallis test. A value of *p* < 0.05 was considered statistically significant.

The detailed methodological workflow, including the experimental protocol, solution preparation, and analytical procedures, is schematically illustrated in Figure 2.

## 3. Results

SEM examination revealed surface coverage and deposit-like surface features with varying morphological characteristics in the groups treated with remineralizing materials compared with the demineralized enamel surface.

- Positive Control Group: The enamel surface exhibited a relatively homogeneous and regular morphology, with preservation of surface integrity (Figure 3A).
- Negative Control Group: Pronounced porous areas, irregular pits, and disruption of surface integrity were observed on the enamel surface (Figure 3B).
- Fluoride Group: Deposit-like surface features covered a large portion of the enamel surface, with marked coverage of porous areas and a more regular surface morphology (Figure 3C).
- CPP-ACP Group: Partial coverage of porous areas and deposit-like surface features were observed; however, these features exhibited a heterogeneous distribution (Figure 3D).
- Propolis Group: Compared with the negative control group, porous areas were less pronounced, and deposit-like surface features were observed in some regions of the surface (Figure 3E).
- Ginger Group: Porous areas were largely covered, and a relatively homogeneous surface appearance with deposit-like features was observed. Among the herbal dentifrice groups, the ginger group exhibited a more regular surface morphology (Figure 3F).
- Aloe Vera Group: Porous areas were partially covered, with deposit-like surface features observed on the surface; however, some microstructural irregularities and linear crack-like formations remained evident (Figure 3G).
- Rosemary Group: Compared with the negative control group, surface irregularities were less pronounced and deposit-like surface features were observed in some areas; however, the surface morphology remained heterogeneous (Figure 3H).

**Figure 3:**
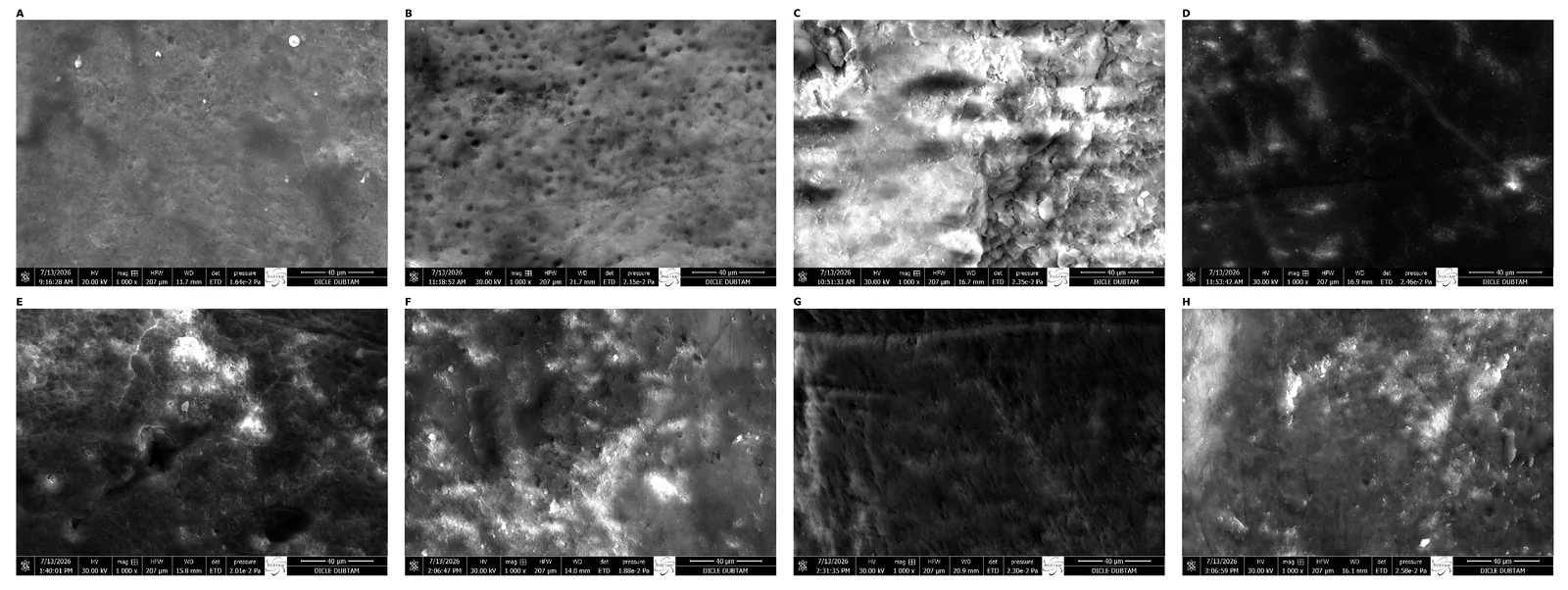
Representative SEM micrographs of the experimental groups at ×1000 magnification (scale bar: 40 µm): (**A**) positive control/sound enamel; (**B**) negative control/demineralized enamel; (**C**) fluoride; (**D**) CPP-ACP; (**E**) propolis; (**F**) ginger; (**G**) aloe vera; and (**H**) rosemary.

### EDX Elemental Analysis Findings

SEM–EDX analysis was used to evaluate the weight (wt%) and atomic (at%) percentages of calcium (Ca), phosphorus (P), and oxygen (O), as well as the Ca/P ratios, across the experimental groups. Effect sizes for between-group comparisons were calculated, and the corresponding η^2^ and ε^2^ values, together with the elemental analysis results and statistical comparisons, are presented in Table 3.

- **Calcium (Ca):** No statistically significant differences were observed among the groups in either Ca wt% or Ca at% values (*p* = 0.085 and *p* = 0.077, respectively). Numerically, the highest mean Ca wt% value was observed in the ginger group, whereas the highest mean Ca at% value was observed in the aloe vera group; however, these numerical differences were not statistically significant.
- **Phosphorus (P):** Statistically significant differences were observed among the groups for both P wt% and P at% values (*p* = 0.008 and *p* = 0.005, respectively). Post hoc analysis for P wt% revealed a significant difference only between the negative control and fluoride groups (adjusted *p* = 0.023). For P at%, statistically significant pairwise differences were identified between the negative control and fluoride groups (*p* = 0.025), the negative control and rosemary groups (*p* = 0.022), the fluoride and ginger groups (*p* = 0.035), and the ginger and rosemary groups (*p* = 0.030).
- **Oxygen (O):** Statistically significant differences were observed among the groups for both O wt% and O at% values (*p* = 0.022 and *p* = 0.020, respectively). However, post hoc pairwise comparisons did not reveal any statistically significant differences for either O wt% or O at% after adjustment for multiple comparisons (all adjusted *p* > 0.05).
- **Ca/P Ratio:** No statistically significant differences were observed among the groups in either the atomic or weight Ca/P ratios (*p* = 0.138 and *p* = 0.126, respectively). The fluoride and aloe vera groups showed the highest numerical mean Ca/P values, whereas the negative control group showed the lowest values; however, these numerical differences were not statistically significant and should not be interpreted as evidence of differences in remineralization efficacy.

The distribution of the mean Ca, P, O, and Ca/P values across the experimental groups is visualized as a standardized heatmap in Figure 4.

## 4. Discussion

The limited number of studies simultaneously comparing commercial pediatric dentifrices containing ginger, propolis, aloe vera, and rosemary with fluoride- and CPP-ACP-containing products within the same experimental model represents an important contribution of the present study. Based on the EDX findings, the null hypothesis (H0) was partially rejected. Although no statistically significant differences were observed among the groups in Ca wt%, Ca at%, or Ca/P ratios (*p* > 0.05), significant between-group differences were detected in both P and O elemental percentages (*p* < 0.05). These findings indicate differences in the relative surface elemental composition among the tested groups; however, they should not be interpreted as direct evidence of differences in absolute mineral gain or remineralization efficacy. The SEM findings were evaluated qualitatively and were therefore not used for statistical testing of the null hypothesis.

In the present study, the effects of fluoride- and CPP-ACP-containing products and commercial pediatric dentifrices containing propolis, ginger, aloe vera, and rosemary on artificially demineralized primary enamel were comparatively evaluated using SEM-EDX analysis and a 7-day pH-cycling model [20]. Buzalaf et al. [20] and ten Cate and Duijsters [21] reported that pH-cycling models provide a laboratory-based simulation of the dynamic acid challenges and buffering processes occurring in the oral environment. Thaveesangpanich et al. [22] indicated that because primary tooth enamel undergoes demineralization more rapidly, prolonged pH-cycling protocols may lead to excessive tissue loss resembling erosion; accordingly, a 7-day pH-cycling protocol was selected in the present study. Tiwari et al. [15] emphasized that applying dentifrices in the form of a *slurry* both simulates salivary dilution and provides reliable methodological standardization. The European Academy of Paediatric Dentistry (EAPD) recommends the use of dentifrices containing 1450 ppm fluoride for children aged 6 years and older [6]. Accordingly, a commercial dentifrice containing 1450 ppm fluoride in its undiluted formulation (Sensodyne ProMine) was selected as the fluoride-containing reference product. However, as all dentifrices were applied experimentally as standardized 1:3 dentifrice–water slurries, the stated 1450 ppm fluoride concentration refers to the original commercial formulation rather than the experimental slurry.

Regarding the SEM findings, Huang et al. [23] and Mutlu [18] demonstrated demineralization-induced changes in surface morphology during SEM examination of artificial initial enamel lesions. Thimmaiah et al. [24] and Hamed et al. [25] reported that remineralizing agents can occlude microporosities on demineralized surfaces, resulting in a more regular surface morphology. Vyavhare et al. [26], Hoffman et al. [27], and Karlinsey et al. [28] reported the formation of compact, acid-resistant calcium fluoride-like mineral deposits on the enamel surface following fluoride application. Svanishvili et al. [29] indicated that mineral deposition in fluoride-based protocols may exhibit regional variations across the enamel surface. Vyavhare et al. [26] reported that CPP-ACP exhibits remineralization potential in initial enamel lesions, although its efficacy may vary when compared with fluoride-containing approaches. This finding is consistent with the present study, in which CPP-ACP was applied for a standardized contact time of 4 min rather than the prolonged contact period recommended by the manufacturer, and the formulation was fluoride-free.

When the herbal agents were evaluated morphologically, Hassan et al. [30], Bilgin et al. [31], and Hossam [32] reported that ginger extracts markedly covered porous areas and produced substantial surface improvement comparable to that achieved with fluoride. Al Haddad et al. [33] reported that aloe vera-containing dentifrices reduced surface porosities and enhanced mineral coverage, although the resulting surface morphology differed from that observed with fluoride. Ali et al. [34] and Zaleh et al. [35] demonstrated that experimental nano-propolis-based approaches may exert favorable effects on the remineralization of artificial white spot lesions, whereas Amalina et al. [36] reported that although propolis reduced surface porosity, it did not completely restore the homogeneous appearance characteristic of sound enamel. Hassan et al. [30] and Hossam [32] further reported that rosemary contributed to tissue repair by providing partial surface coverage of demineralized areas.

In the EDX analyses, no statistically significant differences were observed among the groups in either Ca percentages or Ca/P ratios (*p* > 0.05). Because EDX provides relative elemental composition within the analyzed surface region, these findings should be interpreted as surface compositional measurements rather than direct evidence of absolute mineral gain or remineralization efficacy. Similarly, Svanishvili et al. [29] and Orilisi et al. [37] reported no clear statistically significant superiority among the evaluated groups in terms of EDX-derived calcium-related measurements following remineralization protocols. Nevertheless, the fluoride and aloe vera groups showed the highest numerical mean Ca/P ratios, while the highest numerical Ca values were observed in the ginger and aloe vera groups. As these differences were not statistically significant, they should be regarded solely as descriptive numerical variations and should not be interpreted as evidence of superior mineral gain or remineralization efficacy.

At first glance, the numerically higher Ca and *p* values observed in the negative control group compared with the positive control group may appear inconsistent with the mineral loss expected following demineralization. However, EDX analysis determines the relative percentages of elements within a limited microarea of the enamel surface and does not directly reflect the total mineral loss occurring within the subsurface region of an initial caries lesion. During demineralization, the removal of other surface constituents, such as organic matrix components and carbonate, may have increased the relative proportion of the remaining Ca and P within the total measured elements. Furthermore, because EDX is a surface-sensitive and localized analytical technique, mineral loss occurring within the lesion body may not have been fully reflected in the measured surface composition. Therefore, the higher Ca and P percentages observed in the negative control group should not be interpreted as actual mineral gain but rather in the context of the relative and surface-focused nature of EDX analysis.

Thimmaiah et al. [24] emphasized that the Ca/P ratio, rather than individual mineral concentrations alone, should be considered when interpreting mineralization quality. Al Haddad et al. [33] reported that both fluoride and aloe vera applications increased the Ca/P ratio. Keskin et al. [38] and Mutlu et al. [18] suggested that elemental changes should be evaluated in terms of the balance between Ca and P. Accordingly, the relatively high phosphorus value observed in the negative control group may reflect differences in the relative surface elemental composition after the acid challenge rather than preservation or gain of enamel mineral. Hsu et al. [39] demonstrated that elemental composition may provide complementary information regarding the mineral structure when evaluating enamel mineral phases.

Although statistically significant differences were observed among the groups in phosphorus and oxygen values, changes in these elements should not be regarded as direct indicators of remineralization when considered independently. Because EDX analysis reflects the relative distribution of elements within the examined microarea, changes in the percentage of one element may also be influenced by the relative proportions of other elements. In particular, oxygen may originate not only from the enamel mineral phase but also from various oxygen-containing compounds present on the surface; therefore, an increase in O percentage cannot be directly interpreted as hydroxyapatite formation or mineral gain. Similarly, changes in phosphorus content should be interpreted together with calcium. Consequently, the statistically significant differences in P and O values observed in the present study were not considered independent indicators of superior remineralization but rather complementary indicators of changes in elemental composition, interpreted together with Ca content, the Ca/P ratio, and the surface micromorphological findings observed by SEM.

Acar Özlü and Tuna İnce [9] and Anwar et al. [40] reported that herbal agents inhibit *Streptococcus mutans* biofilm formation and limit demineralization through their polyphenolic and flavonoid constituents. Harman and Akleyin [17] evaluated the remineralization potential of herbal agents, including propolis, grape seed extract, cocoa butter, rosemary oil, and ginger oil, on primary tooth enamel and reported that these agents may support mineral gain. Similarly, Gümüş et al. [41] reported that natural formulations containing rosemary oil, ginger–honey, and grape seed extract exhibited remineralization potential in initial enamel lesions. However, whereas herbal agents in these studies were evaluated in the form of oils, extracts, or natural mixtures, the agents investigated in the present study were evaluated as components of commercially available pediatric dentifrice formulations.

Ranjana et al. [16] and Altınsoy and Ceyhan [42] demonstrated that herbal agents may also exhibit remineralization potential when incorporated into commercial dentifrice formulations. Nevertheless, the effects observed in the present study may have been influenced not only by the herbal extracts themselves but also by other components of the commercial formulations. Specifically, the rosemary-containing product included hydroxyapatite, calcium carbonate, and xylitol; the propolis-containing product included calcium carbonate; and the ginger- and aloe vera-containing products included xylitol. Xylitol has been reported to support the remineralization process [43]. Therefore, the elemental and morphological changes observed in the herbal groups cannot be attributed solely to the respective herbal extracts, and the potential contribution of the overall product formulations should be considered.

In young children, inadvertent swallowing of dentifrice and the risk of dental fluorosis associated with excessive fluoride exposure highlight the importance of using fluoride-containing dentifrices at age-appropriate concentrations and amounts [6]. In the present study, the fluoride group exhibited a comparatively homogeneous surface morphology on SEM, while the ginger- and aloe vera-containing products showed distinct surface morphological patterns and numerical variations in EDX-derived elemental composition. However, because the observed Ca and Ca/P differences were not statistically significant and the tested products were multi-ingredient commercial formulations, these findings should not be interpreted as evidence of superior remineralization efficacy attributable to any individual active ingredient.

### 4.1. Strengths of the Study

Standardization of the demineralization and pH-cycling conditions across the relevant experimental groups, as well as the material application times across the treatment groups, ensured methodological consistency. In young children, inadvertent swallowing of dentifrice and the risk of dental fluorosis associated with excessive fluoride intake represent important clinical concerns, while concerns regarding fluoride use and the growing interest in naturally derived products have encouraged the use of alternative products [44]. The present study contributes to the pediatric dentistry literature by directly comparing the reference agent fluoride and CPP-ACP with herbal pediatric dentifrices containing propolis, ginger, aloe vera, and rosemary within the same experimental model. The use of readily available commercial products enhances the applicability of the findings to future clinical and in situ studies. Furthermore, the combined use of SEM for qualitative assessment of surface micromorphology and EDX for evaluation of relative surface elemental composition (Ca, P, O, and Ca/P ratios) enabled complementary assessment of morphological and compositional changes in the enamel surface.

### 4.2. Limitations of the Study

Because the study was conducted under laboratory conditions, intraoral biological factors such as natural salivary flow, acquired pellicle formation, microbial biofilm, and masticatory forces could not be fully simulated. The sample size of 10 specimens per group may have limited the ability to detect statistically significant differences when relatively small numerical differences existed among the groups. Although five different microareas were evaluated for each specimen, the localized nature of EDX measurements may limit their ability to fully represent the elemental composition of the entire enamel surface. In addition, the Au–Pd conductive coating required for SEM examination may influence the detected X-ray spectrum; although Au–Pd peaks were excluded from quantitative elemental analysis, the use of a conductive coating should be considered when interpreting the EDX-derived relative elemental composition.

Moreover, complementary quantitative techniques such as surface microhardness testing, transverse microradiography, or profilometry were not employed in the present study. Therefore, the SEM-EDX findings should be interpreted primarily as qualitative morphological and relative surface compositional changes rather than as direct quantitative evidence of mineral gain. The use of commercial dentifrice formulations rather than pure herbal extracts represents an important limitation of the present study. Consequently, it is difficult to determine whether the observed effects resulted specifically from the bioactive properties of propolis, aloe vera, or rosemary extracts or from the synergistic effects of auxiliary remineralizing components in the dentifrice base formulations, such as inorganic calcium salts (e.g., calcium carbonate), hydroxyapatite, or xylitol. Moreover, because the evaluated herbal products were formulations approved for individuals aged 6 years and older, the findings should not be directly generalized to younger age groups.

The application protocol used for the CPP-ACP-containing GC Tooth Mousse represents another methodological limitation. According to the manufacturer’s recommendations, this material is intended to be applied undiluted and to remain in contact with the enamel surface for a longer period as a topical cream. However, to standardize application conditions across the experimental groups, CPP-ACP was prepared as a 1:3 slurry with distilled water and applied for 4 min, consistent with the protocol used for the other dentifrices. This dilution and relatively short contact time may have influenced the surface availability and observed performance of the CPP-ACP-containing product under the present experimental conditions, and the findings should therefore not be considered representative of the manufacturer-recommended clinical application protocol.

## 5. Conclusions

In this in vitro study, SEM examination revealed qualitative differences in surface micromorphology among the tested groups, while EDX analysis demonstrated significant between-group differences in P and O percentages. However, no statistically significant differences were observed in Ca percentages or Ca/P ratios (*p* > 0.05). The fluoride group exhibited a comparatively homogeneous surface morphology, while the ginger-containing dentifrice also showed a relatively homogeneous surface appearance among the commercial herbal formulations. Although numerical variations in Ca and Ca/P values were observed among the groups, these differences were not statistically significant and should not be interpreted as evidence of superior mineral gain or remineralization efficacy. Given the relative and surface-focused nature of EDX analysis and the multi-ingredient composition of the tested commercial products, the findings should be interpreted as morphological and surface compositional changes rather than direct evidence of remineralization attributable to individual active ingredients. Further in situ and in vivo studies are required to determine the clinical relevance of these findings.

## Figures and Tables

**Figure 1:**
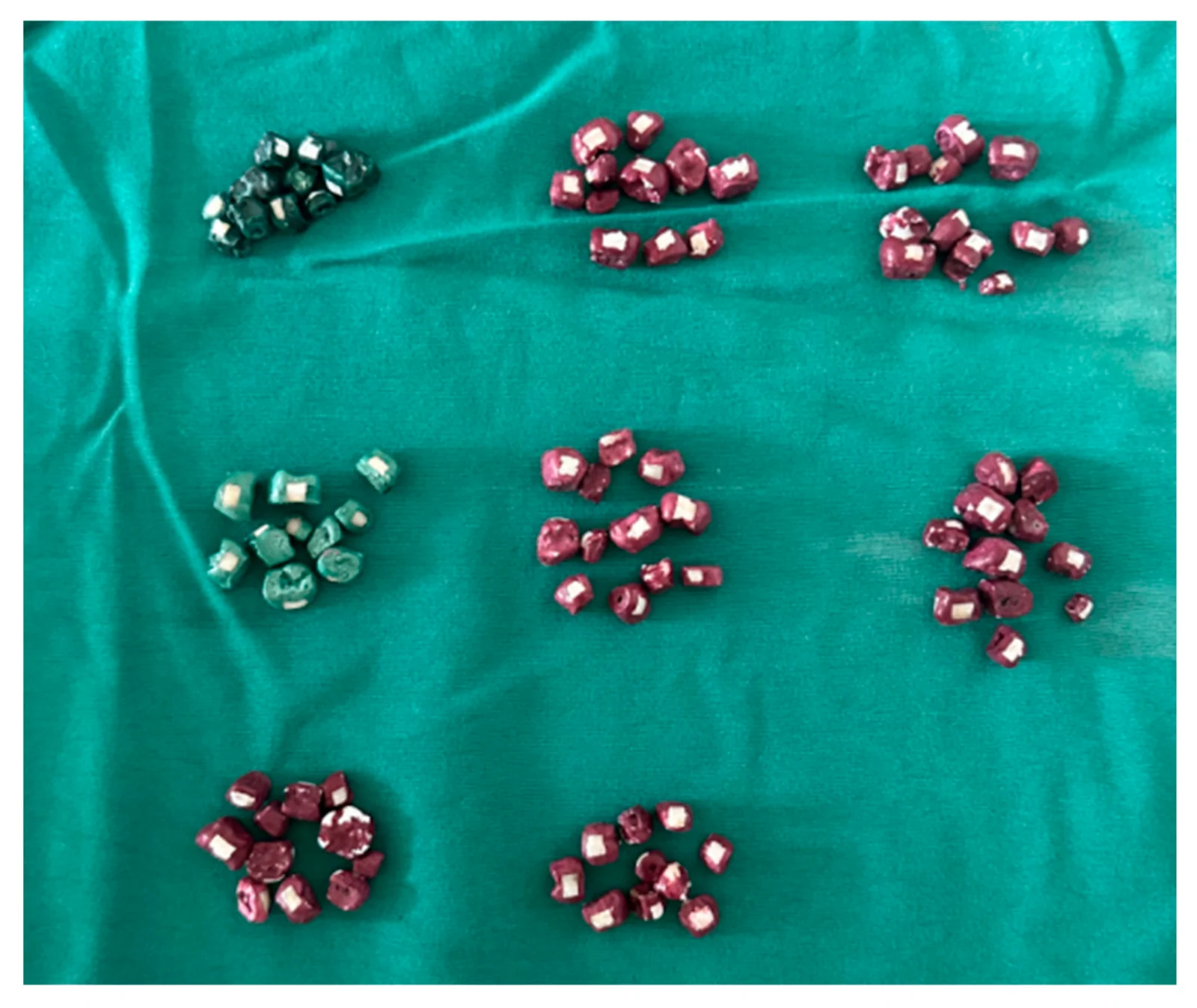
Overview of the experimental groups and tooth specimens.

**Figure 2:**
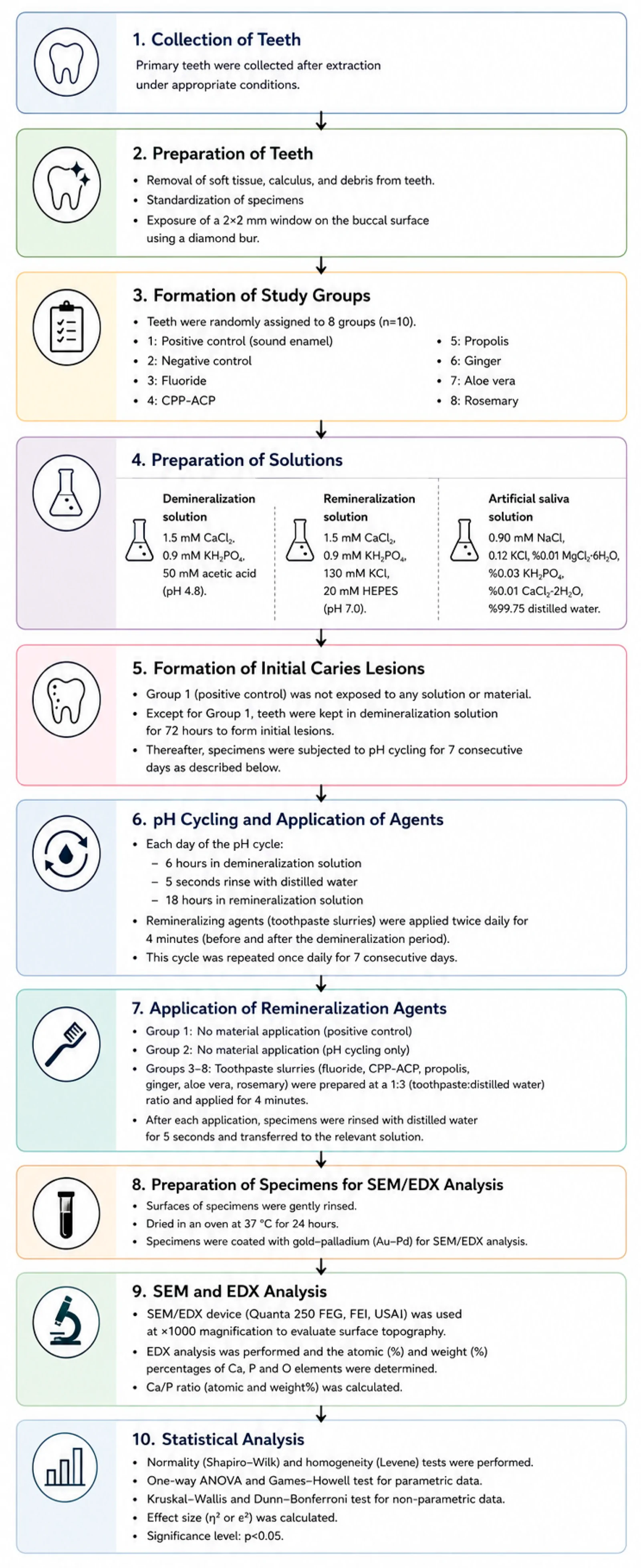
Experimental workflow illustrating the preparation of primary tooth specimens, formation of artificial initial enamel lesions, pH cycling, remineralization protocol, and analytical procedures.

**Figure 4:**
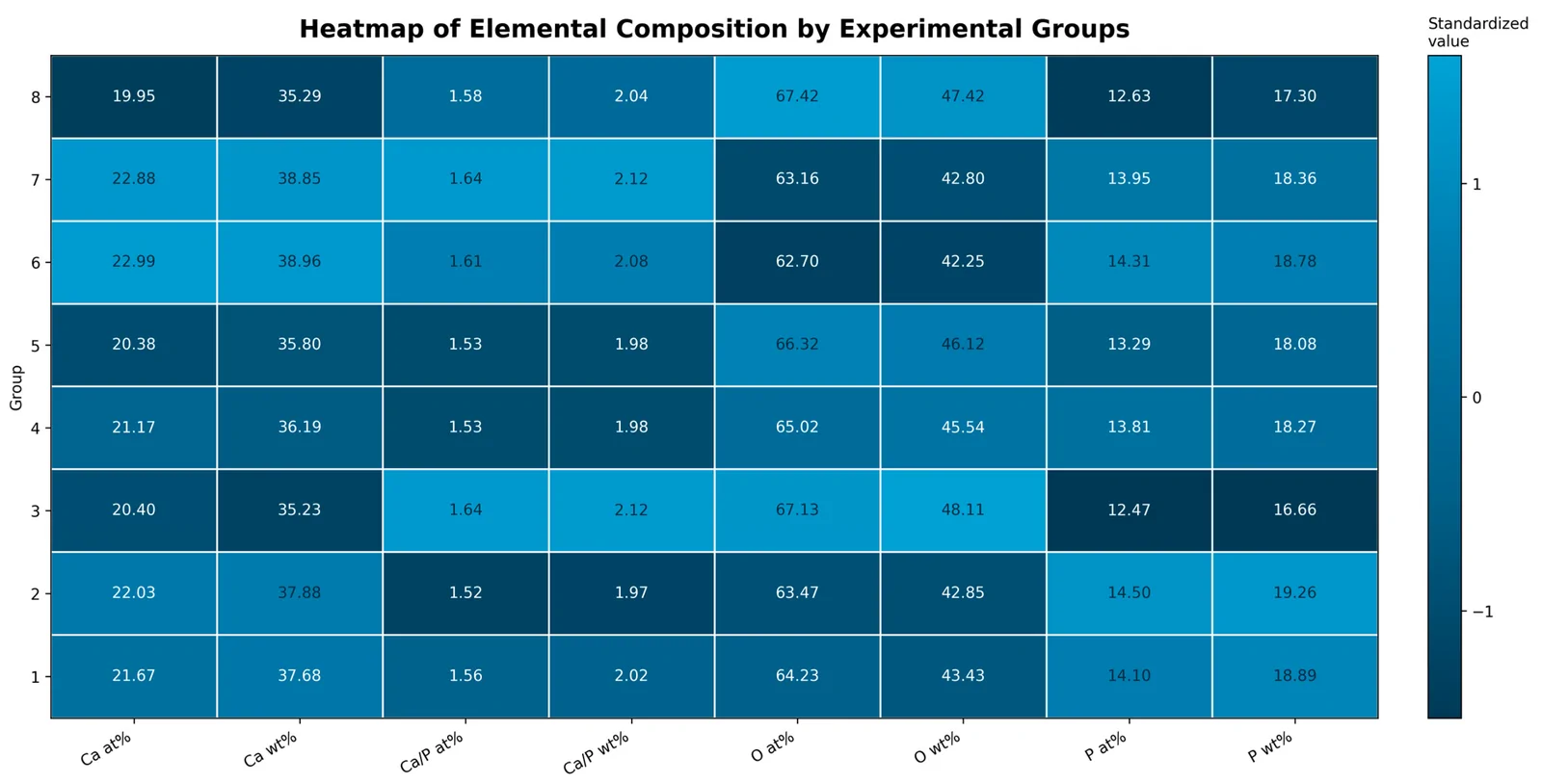
Standardized heatmap showing the mean EDX-derived Ca, P, and O weight (wt%) and atomic (at%) percentages and Ca/P ratios across the experimental groups. Group 1: positive control; Group 2: negative control; Group 3: fluoride; Group 4: CPP-ACP; Group 5: propolis; Group 6: ginger; Group 7: aloe vera; Group 8: rosemary.

**Table 1:** Commercial products used in the study and their compositions.

Product	Composition
Sensodyne Promine Kids 6–12 Years	Aqua, Sorbitol, Hydrated Silica, Glycerin, PEG-6, Cocamidopropyl Betaine, Xanthan Gum, Aroma, Sodium Saccharin, Sodium Fluoride (1450 ppm F^−^), Limonene, Sodium Hydroxide, CI 42090
GC Tooth Mousse	Pure Water, Glycerol, RECALDENT™ (CPP-ACP), D-Sorbitol, CMC-Na, Propylene Glycol, Silicon Dioxide, Titanium Dioxide, Xylitol, Phosphoric Acid, Flavoring, Zinc Oxide, Sodium Saccharin, Ethyl p-Hydroxybenzoate, Magnesium Oxide, Guar Gum, Propyl p-Hydroxybenzoate, Butyl p-Hydroxybenzoate
DK Dent Propolis	Aqua, Calcium Carbonate, Sorbitol, Hydrated Silica, Glycerin, Polysorbate-20, Clay, Cocamidopropyl Betaine, Propolis Extract, Aroma, Disodium Phosphate, Xanthan Gum, Stevia Rebaudiana Leaf Powder, Allcloxa, Propylene Glycol, Menthol, Sodium Benzoate
Eyüp Sabri Tuncer Ginger	Aqua, Hydrated Silica, Sorbitol, Glycerin, Xylitol, Polysorbate 20, Disodium Phosphate, Xanthan Gum, Aroma, *Zingiber Officinale* (Ginger) Root Oil, *Zingiber Officinale* (Ginger) Root Extract, *Eucalyptus Globulus Leaf Oil*, *Mentha Piperita* (Peppermint) Oil, *Cymbopogon martinii* (Palmarosa) Oil, *Pelargonium graveolens* Flower Oil, Potassium Saccharin, Phenoxyethanol, Caprylyl Glycol, Thymol, Tocopherol, Limonene
DK Dent Aloe Vera	Aqua, Hydrated Silica, Glycerin, Xylitol, Xanthan Gum, Cocamidopropyl Betaine, *Aloe barbadensis* Leaf Extract, *Eugenia caryophyllus* (Clove) Flower Extract, Clay, Aroma, *Stevia rebaudiana* Leaf Powder, Sodium Benzoate
Ormanya Rosemary	Hydrated Silica, Sorbitol, Aqua, Glycerin, Hydroxyapatite, Cocamidopropyl Betaine, Xylitol, Flavor, CMC, Xanthan Gum, Menthol, Potassium Sorbate, Calcium Carbonate, *Stevia rebaudiana* Leaf Powder, *Mentha piperita* Oil, *Rosmarinus officinalis* Leaf Extract

Product compositions are presented according to the ingredient lists provided by the manufacturers. The fluoride group contained 1450 ppm F^−^ derived from sodium fluoride (NaF). No fluoride-containing ingredients were listed in the manufacturer-reported compositions of the propolis, ginger, aloe vera, or rosemary products. Relative dentin abrasivity (RDA) values were not included in the table because they were not reported by the manufacturers.

**Table 2:** pH-cycling protocol applied to the experimental groups and application steps of the remineralization materials.

Time	Positive Control (No Solution or Material)	Negative Control (pH Cycling Only)	Fluoride Toothpaste	CPP-ACP Cream	Propolis Toothpaste	Ginger Toothpaste	Aloe Vera Toothpaste	Rosemary Toothpaste
18 h	Remineralization solution (pH 7.0)							
5 s	Rinse with distilled water							
4 min	– (No application)	– (No application)	Apply toothpaste slurry *	Apply cream slurry *	Apply toothpaste slurry *	Apply toothpaste slurry *	Apply toothpaste slurry *	Apply toothpaste slurry *
5 s	Rinse with distilled water							
6 h	Demineralization solution (pH 4.8)							
4 min	– (No application)	– (No application)	Apply toothpaste slurry *	Apply cream slurry *	Apply toothpaste slurry *	Apply toothpaste slurry *	Apply toothpaste slurry *	Apply toothpaste slurry *

* Toothpaste-to-water ratio 1:3 (slurry). Applied twice daily (before and after the demineralization period) for 4 min each time.

**Table 3:** Enamel surface elemental distributions of calcium (Ca), phosphorus (P), and oxygen (O), Ca/P ratios, and results of statistical comparisons among the experimental groups.

Group	Ca wt%	Ca at%	P wt%	P at%	O wt%	O at%	Ca/P at%	Ca/P wt%
Positive control	37.68 ± 3.31	21.67 ± 2.40	18.89 ± 2.06	14.10 ± 2.02	43.43 ± 3.74	64.23 ± 3.70	1.56 ± 0.23	2.02 ± 0.30
Negative control	37.88 ± 1.96	22.03 ± 1.42	19.26 ± 1.19	14.50 ± 1.09	42.85 ± 3.01	63.47 ± 2.44	1.52 ± 0.05	1.97 ± 0.07
Fluoride	35.23 ± 4.09	20.40 ± 2.43	16.66 ± 1.99	12.47 ± 1.30	48.11 ± 5.56	67.13 ± 3.36	1.64 ± 0.16	2.12 ± 0.21
CPP-ACP	36.19 ± 3.93	21.17 ± 3.06	18.27 ± 1.56	13.81 ± 1.65	45.54 ± 5.40	65.02 ± 4.67	1.53 ± 0.07	1.98 ± 0.09
Propolis	35.80 ± 4.21	20.38 ± 2.94	18.08 ± 1.40	13.29 ± 1.27	46.12 ± 5.43	66.32 ± 4.13	1.53 ± 0.11	1.98 ± 0.14
Ginger	38.96 ± 3.33	22.99 ± 2.58	18.78 ± 1.01	14.31 ± 0.94	42.25 ± 3.73	62.70 ± 3.23	1.61 ± 0.15	2.08 ± 0.19
Aloe vera	38.85 ± 3.51	22.88 ± 2.85	18.36 ± 0.80	13.95 ± 0.90	42.80 ± 3.98	63.16 ± 3.63	1.64 ± 0.13	2.12 ± 0.16
Rosemary	35.29 ± 3.64	19.95 ± 2.58	17.30 ± 1.14	12.63 ± 1.10	47.42 ± 4.56	67.42 ± 3.57	1.58 ± 0.11	2.04 ± 0.15
Test statistic	F = 1.881	H = 12.805	H = 18.961	F = 3.257	F = 2.538	H = 16.579	H = 11.004	H = 11.304
*p* value	*p* = 0.085	0.077	**0.008**	**0.005**	**0.022**	**0.020**	0.138	0.126
Effect size	η^2^ = 0.155	ε^2^ = 0.081	ε^2^ = 0.166	η^2^ = 0.241	η^2^ = 0.198	ε^2^ = 0.133	ε^2^ = 0.056	ε^2^ = 0.060

Data are presented as mean ± standard deviation. η^2^: eta-squared effect size for one-way analysis of variance (ANOVA); ε^2^: epsilon-squared effect size for the Kruskal–Wallis test; F: test statistic for one-way analysis of variance (ANOVA); H: Kruskal–Wallis test statistic; CPP-ACP: casein phosphopeptide–amorphous calcium phosphate; wt%: weight percentage; at%: atomic percentage. Statistically significant *p* values are shown in bold (*p* < 0.05). Post hoc analyses revealed significant pairwise differences for P wt% (negative control vs. fluoride, adjusted *p* = 0.023) and P at% (negative control vs. fluoride, *p* = 0.025; negative control vs. rosemary, *p* = 0.022; fluoride vs. ginger, *p* = 0.035; ginger vs. rosemary, *p* = 0.030). Although the overall comparisons for O wt% and O at% showed statistically significant differences, no significant pairwise differences were identified after adjustment for multiple comparisons.

## Data Availability

The data supporting the findings of this study are available within the article and its Supplementary Materials. The Supplementary Materials include the specimen-level EDX dataset used for the reported analyses, including the normalized Ca, P, and O weight and atomic percentages and the corresponding calculated Ca/P ratios.

## Supplementary Materials

The following supporting information can be downloaded at https://www.mdpi.com/article/10.3390/jfb17100494/s1. Supplementary Data S1: Specimen-level EDX dataset including source and proportionally normalized Ca, P, and O weight (wt%) and atomic (at%) percentages and calculated Ca/P ratios; Figure S1: Representative EDX spectra of the experimental groups.
